# Supplementary material for: Multiscale real time and high sensitivity ion detection with complementary organic electrochemical transistors amplifier
Source: Nat Commun. 2020 Jul 27;11:3743. doi: 10.1038/s41467-020-17547-0 (PMC7385487; doi:10.1038/s41467-020-17547-0)
Supplement: Supplementary file 1 — Supplementary Information [file 41467_2020_17547_MOESM1_ESM.pdf]

SUPPLEMENTARY INFORMATION

Multiscale real time and high sensitivity ion detection with complementary organic electrochemical transistors amplifier

Paolo Romele[1], Paschalis Gkoupidenis[2], Dimitrios Koutsouras[2], Katharina Lieberth[2], Zsolt M. Kovács-Vajna[1], Paul W. M. Blom[2] & Fabrizio Torricelli[1]

[1]University of Brescia, Department of Information Engineering, via Branze 38, 25123 Brescia, Italy.

[2]Max Planck Institute for Polymer Research, Ackermannweg 10, Mainz 55128, Germany.

Correspondence and requests for materials should be addressed to F.T. (email: fabrizio.torricelli@unibs.it)

SUPPLEMENTARY FIGURES

Pag. 2 Supplementary Figure 1: Typical transfer characteristics of p-type and n-type transistors.
Pag. 3 Supplementary Figure 2: Transient response of the device.
Pag. 4 Supplementary Figure 3: Relationship between amplification and ion concentration.
Pag. 5 Supplementary Figure 4: Selective ion detection.
Pag. 6 Supplementary Figure 5: Blood serum selective ion detection.
Pag. 7 Supplementary Figure 6: Andrea Mantegna and the multiscale approach.
Pag. 8 Supplementary Figure 7: Image of the ion sensing device.
Pag. 9 Supplementary Figure 8: transistors output characteristics.

SUPPLEMENTARY NOTES

Pag. 10 Supplementary Note 1: derivation of the OECT complementary amplifier transition voltage and amplification
Pag. 12 Supplementary Note 2: amplification dependence on *c* and derivation of the minimum detectable concentration variation

Supplementary References

SUPPLEMENTARY FIGURES

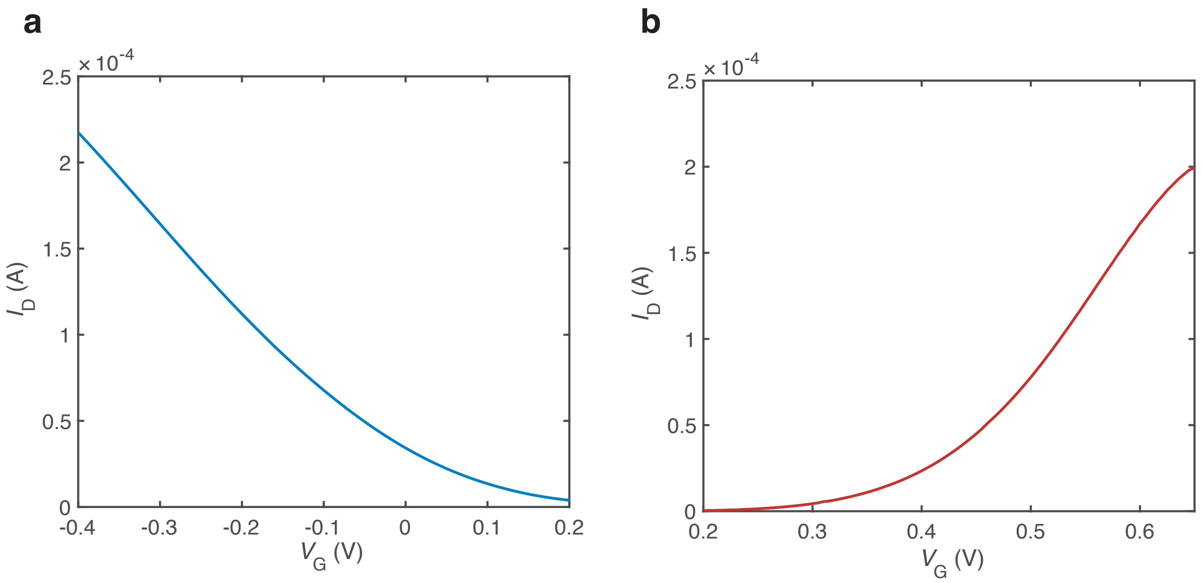

Supplementary Figure 1: Typical transfer characteristics of p-type and n-type transistors. a Transfer characteristic of a p-type PEDOT:PSS organic electrochemical transistor measured at V_D = -0.1 V and c = 10^{-1} M. By applying a positive gate voltage (V_G), cations are injected into the channel, reducing its hole concentration and lowering the drain current. Analogously, when a negative bias is applied, anions drift into the polymer, the hole concentration increases and this results in a larger I_D. **b** Transfer characteristic of an n-type BBL OECT measured with V_D = 0.1 V and c = 10^{-1} M. By increasing V_G, the injection of cations into the polymer increases the electron concentration and, as a consequence, I_D increases.

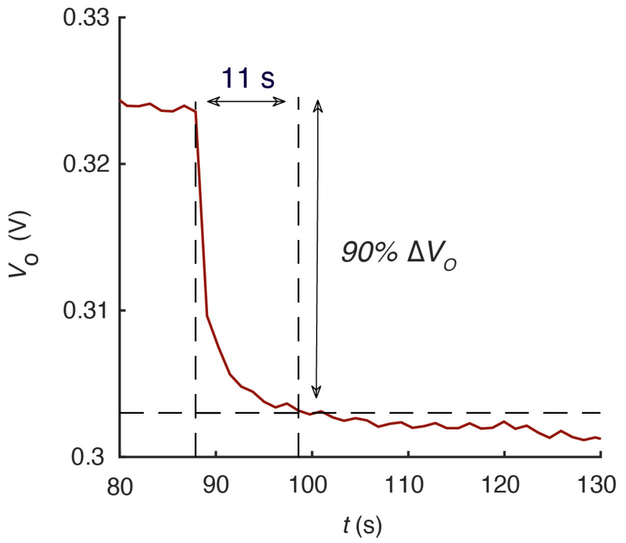

Supplementary Figure 2: Transient response of the device. Output voltage measured over time when *c* increases from 54 10^{-5} to 61 10^{-5}.

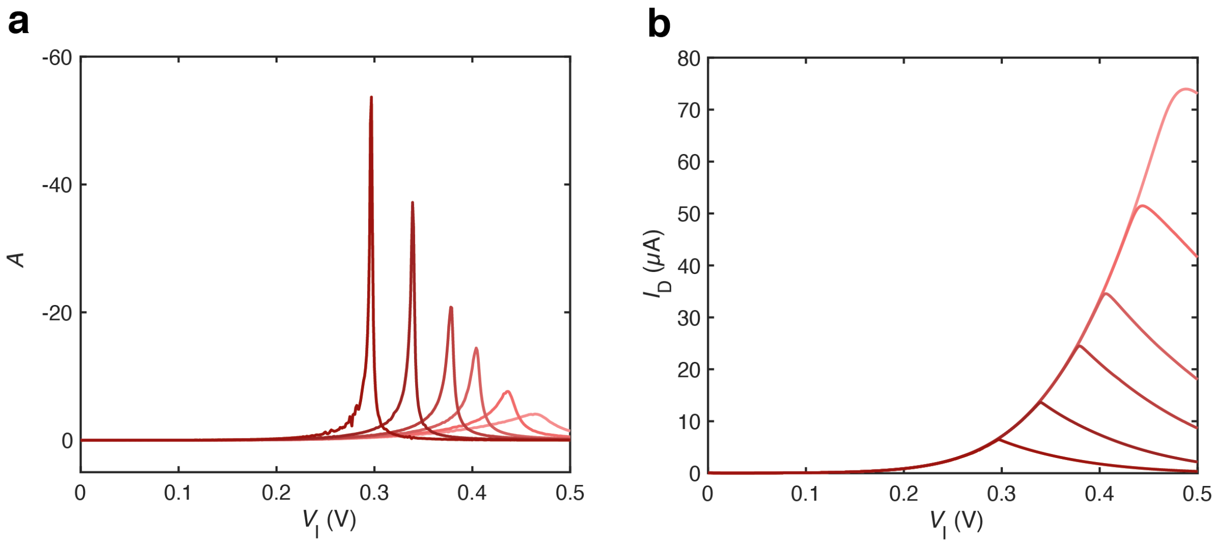

Supplementary Figure 3: Relationship between amplification and ion concentration. a Measured amplification *A* of the complementary organic electrochemical transistor amplifier at various ion concentrations. From the rightmost to the leftmost characteristic the ion concentration is equal to [10^{-5}, 10^{-4}, 10^{-3}, 10^{-2}, 10^{-1}, 1] M. **b** Measured I_D of the OECT complementary amplifier at various ion concentrations. From the lightest to the darkest characteristic the ion concentration is equal to [10^{-5}, 10^{-4}, 10^{-3}, 10^{-2}, 10^{-1}, 1] M.

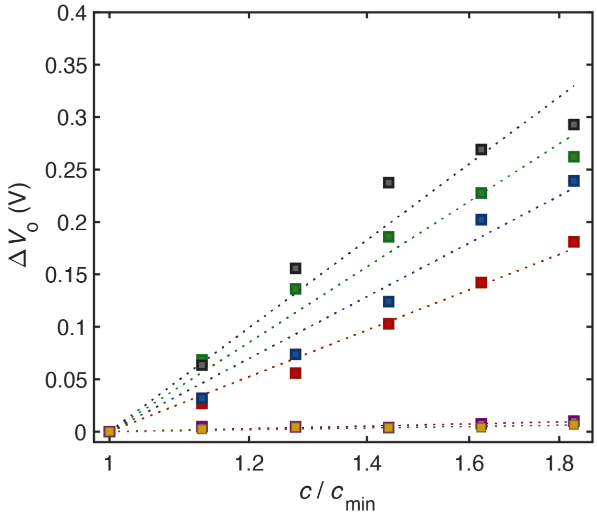

Supplementary Figure 4: Selective ion detection. Measured (symbols) output voltage variation $\Delta V_O = V_O(c) - V_O(c_{min})$ as a function of c/c_{min}. The organic electrochemical transistor complementary amplifier is selective to K^+ ions. Red, blue, green and grey symbols are measured with $c^{K+}{}_{min}$ = [5.4 x 10^{-4} 5.4 x 10^{-3} 5.4 x 10^{-2} 5.4 x 10^{-1}] M, respectively. Violet symbols are the control experiment measured with $c^{Na+}{}_{min}$ = 5.4 10^{-2} M, brown symbols are the control experiment measured with $c^{Ca2+}{}_{min}$ = 5.4 10^{-2} M. Dotted lines are guides for the eye.

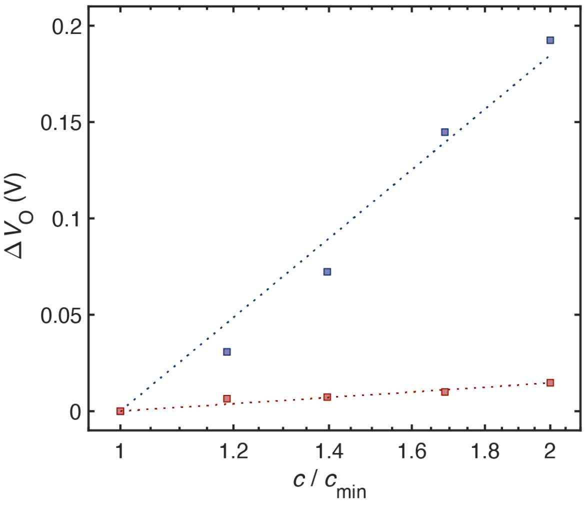

Supplementary Figure 5: Blood serum selective ion detection. Measured (symbols) output voltage variation $\Delta V_O = V_O(c)$ - $V_O(c_{min})$ as a function of c/c_{min} in blood serum sample. The organic electrochemical transistor complementary amplifier is selective to K^+ ions. Blue symbols are measured at $c^{K+}_{min} = 4.8\ 10^{-3}$ M. Red symbols are the control experiment measured at $c^{Na+}_{min} = 1.36\ 10^{-1}$ M. Dotted lines are guides for the eye.

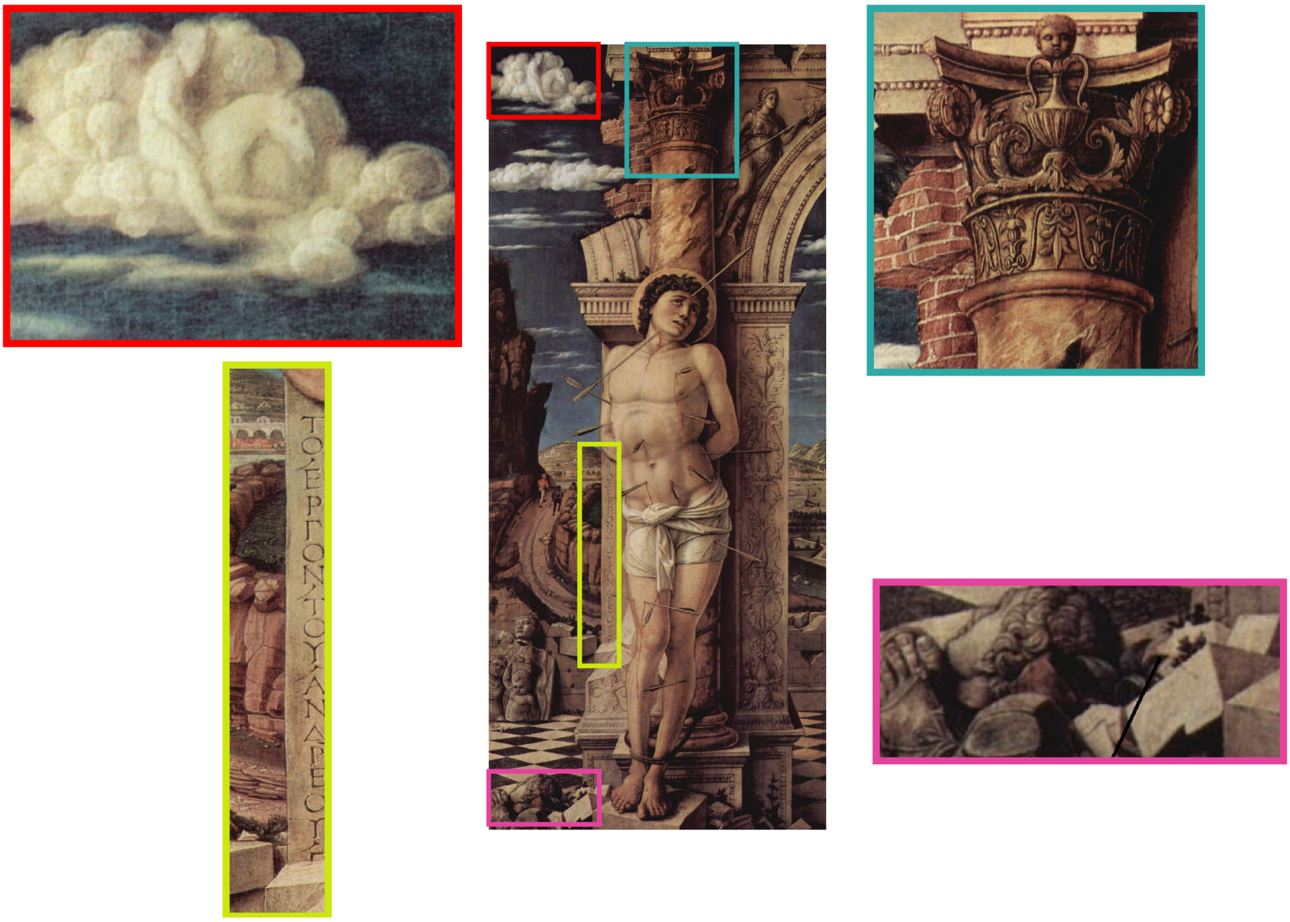

Supplementary Figure 6: Andrea Mantegna and the multiscale approach. The multiscale approach is something we naturally use in everyday life. For example, when admiring a masterpiece of art, we usually have a first look from the distance, appreciating the global context (i.e. the central picture of the figure), then we move closer to capture the details (i.e. zoom in the corners). Here, the central picture displays the saint Sebastian, painted by Andrea Mantegna around in the year 1457 in Padua. At that time, Europe was frequently struck by plague epidemics, and Mantegna himself got sick. In 1457 the pestilence ended in Padua and Mantegna miraculously recovered. Saint Sebastian is usually represented as patron saint of plague victims and this painting was probably commissioned to him to celebrate the end of the epidemics. If we have a closer look to the details, a rider with a scythe is present in the clouds at the top left corner. The rider has been interpreted as Saturn, the Roman god, associated to the Greek Cronus, identified in ancient times as the time that passed by and all left destroyed behind him, and saint Sebastian is invoked against him. Next to saint Sebastian we can notice the signature of the artist in Greek, ΤΟ.ΕΡΓΟΝ.ΤΟΥ.ΑΝΔΡΕΟΥ, "Artwork by Andrea", recalling the classical culture. At the top of the column behind saint Sebastian a Corinthian capital is also recalling Greek culture, as well as the ancient ruins at the saint's feet.

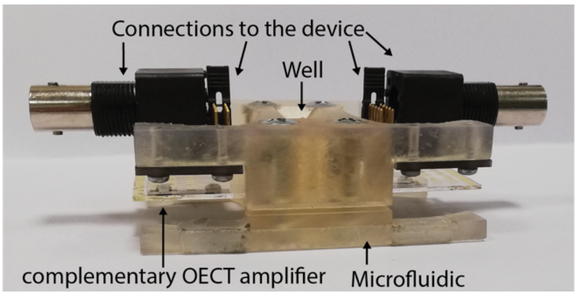

Supplementary Figure 7: Image of the ion sensing device. The fabricated complementary organic electrochemical transistor amplifier is hosted into a custom 3D printed microfluidic that provides reliable electrical connection with the measurement instruments and a well containing the analyte.

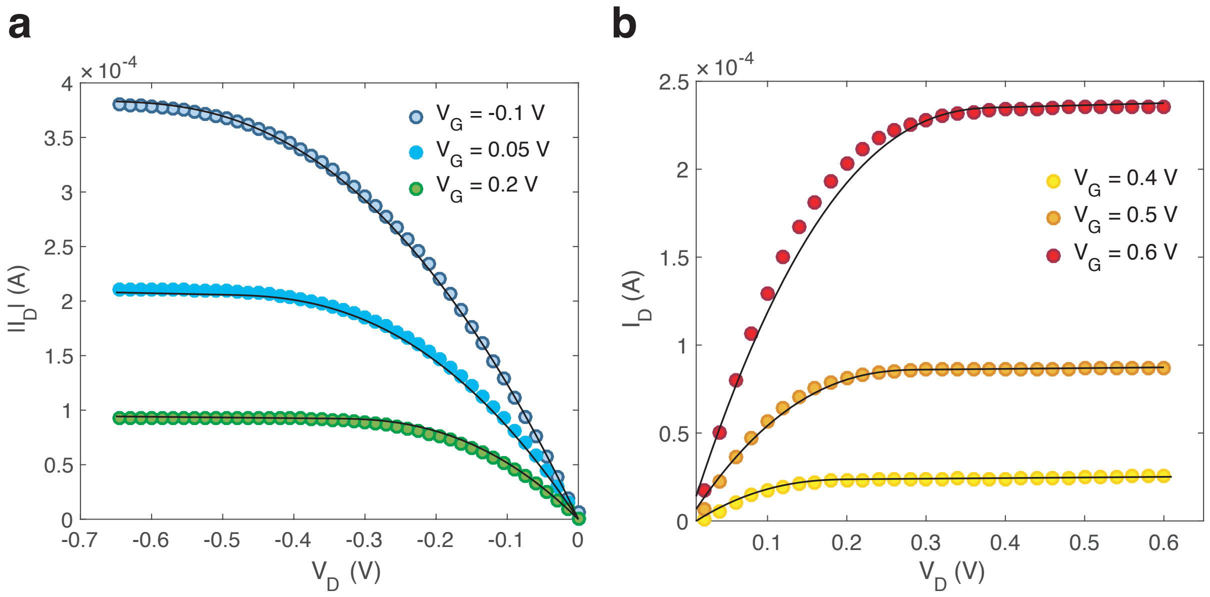

Supplementary Figure 8: transistors output characteristics. a Typical output characteristics I_D-V_D measured (symbols) at several V_G of a p-type PEDOT:PSS organic electrochemical transistor. **b** Typical output characteristics I_D-V_D measured (symbols) at several V_G of a n-type BBL OECT. Full lines are calculated with the OECTs model showed in ref. 1 including the channel length modulation[3].

SUPPLEMENTARY NOTES

Supplementary Note 1: derivation of the OECT complementary amplifier transition voltage and amplification

We define the transition voltage V_M as the input voltage that yields $V_O = V_{DD} / 2$. An analytical formulation for V_M can be obtained by considering the p-type and n-type OECTs currents when $V_I = V_M$ and $V_O = V_{DD} / 2$. We use the Bernards-Malliaras model[1] for the OECTs drain current evaluated in the saturation regime including the ion-concentration dependent threshold voltage[2], and it reads:

$$I_{\mathrm{Dn}} = \frac{\Gamma_{\mathrm{n}}}{2}(V_{\mathrm{I}} - V_{\mathrm{Tn}})^2[1 + \lambda_{\mathrm{n}} V_{\mathrm{O}}] \tag{1}$$

$$I_{\mathrm{Dp}} = \frac{\Gamma_{\mathrm{p}}}{2}\left(V_{\mathrm{DD}} - V_{\mathrm{I}} + V_{\mathrm{Tp}}\right)^2[1 + \lambda_{\mathrm{p}}(V_{\mathrm{DD}} - V_{\mathrm{O}})] \tag{2}$$

where:

$$\Gamma = \frac{Wt}{L}\mu C_{\mathrm{v}} \tag{3}$$

$$V_{\mathrm{T}} = V_{\mathrm{P}} - V_{\mathrm{SH}} \tag{4}$$

where Γ_n, and Γ_p are given by Supplementary Equation (3) and depends on both geometrical and technological parameters, viz. channel width W, length L and thickness t, electron or hole mobility μ, and volumetric capacitance C_v of the channel. The threshold voltage V_{Tn} and V_{Tp} are calculated with Supplementary Equation (4) where V_P is the pinch-off voltage[1] V_{SH} accounts for the voltage shift as a function of the ion concentration c[2], and λ accounts for the channel length modulation[3] ($\lambda \propto g_o$, $g_o = r_o^{-1}$)

V_M can be obtained by equating the p-type and n-type drain current given by Supplementary Equation (1) and Supplementary Equation (2) and solving for $V_I = V_M$, considering that in our OECTs $\lambda_p = \lambda_n$ (Supplementary Figure 8):

$$V_{\mathrm{M}} = \frac{V_{\mathrm{Tn}} + \eta V_{\mathrm{Tp}} + \eta V_{\mathrm{DD}}}{1 + \eta} \tag{5}$$

where $\eta = (\Gamma_p \Gamma_n^{-1})^{1/2}$. It is worth noting that Supplementary Equation (5) is formally the same as the classical switching voltage of a CMOS inverter where $V_O = V_I$, but here the meaning is different because $V_O = V_{DD} / 2$ independently of V_I.

The amplifier gain A can be obtained by considering that the same drain current flows in both the p-type and n-type OECTs. We define the function f as the sum of the p-type and n-type OECT currents, and it reads:

$$f = \frac{\Gamma_{\mathrm{n}}}{2}(V_{\mathrm{I}} - V_{\mathrm{Tn}})^2[1 + \lambda_{\mathrm{n}} V_{\mathrm{O}}] - \frac{\Gamma_{\mathrm{p}}}{2}\left(V_{\mathrm{DD}} - V_{\mathrm{I}} + V_{\mathrm{Tp}}\right)^2[1 + \lambda_{\mathrm{p}}(V_{\mathrm{DD}} - V_{\mathrm{O}})] \tag{6}$$

$A = \partial V_O / \partial V_I$ can be calculated as:

$$A = \left(\frac{df}{dV_{\mathrm{I}}}\right)\left(\frac{df}{dV_{\mathrm{O}}}\right)^{-1} \tag{7}$$

where:

$$\begin{aligned} \frac{df}{dV_{\mathrm{I}}} &= \Gamma_{\mathrm{n}}(V_{\mathrm{I}} - V_{\mathrm{Tn}})[1 + \lambda_{\mathrm{n}} V_{\mathrm{O}}] - \Gamma_{\mathrm{p}}\left(V_{\mathrm{DD}} - V_{\mathrm{I}} + V_{\mathrm{Tp}}\right)\left[1 + \lambda_{\mathrm{p}}(V_{\mathrm{DD}} - V_{\mathrm{O}})\right] = \\ &= g_{\mathrm{mn}}[1 + \lambda_{\mathrm{n}} V_{\mathrm{O}}] + g_{\mathrm{mp}}[1 + \lambda_{\mathrm{p}}(V_{\mathrm{DD}} - V_{\mathrm{O}})] \end{aligned} \tag{8}$$

and

$$\frac{df}{dV_{\mathrm{O}}} = \Gamma_{\mathrm{n}}(V_{\mathrm{I}} - V_{\mathrm{Tn}})^2 \lambda_{\mathrm{n}} + \Gamma_{\mathrm{p}}\left(V_{\mathrm{DD}} - V_{\mathrm{I}} + V_{\mathrm{Tp}}\right)\lambda_{\mathrm{p}} \tag{9}$$

Therefore, the amplification A evaluated at the switching voltage V_M where $V_O = V_{DD} / 2$ results:

$$A(V_{\mathrm{M}}) = \frac{1}{I_{\mathrm{D}}(V_{\mathrm{M}})} \frac{g_{\mathrm{mn}}\left[1+\lambda_{\mathrm{n}}\frac{V_{\mathrm{DD}}}{2}\right] + g_{\mathrm{mp}}\left[1+\lambda_{\mathrm{p}}\frac{V_{\mathrm{DD}}}{2}\right]}{\lambda_{\mathrm{n}}+\lambda_{\mathrm{p}}} \tag{10}$$

Supplementary Note 2: amplification dependence on c and derivation of the minimum detectable concentration variation

The real time sensitivity of the amplifier is given by $S_A = A\, S_M$ (Eq. (3) of the manuscript). Supplementary Figure 3a shows that $A = dV_O/dV_I$ increases with increasing ion concentration, yielding a higher S_A. This can be explained as follows. According to Eq. (4) the amplification is inversely proportional to $I_D(V_M)$, that is the current flowing through the OECTs at $V_I = V_M$. Supplementary Figure 3b shows that $I_D(V_M)$ progressively decreases by increasing the ion concentration. The lower $I_D(V_M)$ can be explained by considering that at larger concentrations the OECTs show a lower threshold voltage V_{Tp}, and this yields a lower drain current when $V_I = V_M$. As a consequence, higher concentrations result in larger A and, in turn, in a larger sensitivity. This allows the complementary OECT amplifier to detect small variations of the ion concentrations even when the total concentration is large. The output voltage variation ΔV_O due to a concentration variation from c_i to c_f reads:

$$\Delta V_{\mathrm{O}} = S_{\mathrm{A}}[\log_{10}(c_{\mathrm{f}}) - \log_{10}(c_{\mathrm{i}})] \tag{11}$$

Solving for c_f/c_i we obtain:

$$\frac{c_{\mathrm{f}}}{c_{\mathrm{i}}} = 10^{\Delta V_{\mathrm{O}}/S_{\mathrm{A}}} \tag{12}$$

Thus resulting in:

$$\Delta c = c_{\mathrm{f}} - c_i = \left(10^{\frac{\Delta V_{\mathrm{O}}}{S_{\mathrm{A}}}} - 1\right) c_{\mathrm{i}} \tag{13}$$

Therefore, when the initial ion concentration is the maximum of the range (viz. $c_i = c_{max}$) the minimum variation Δc_{min} resulting in the minimum measurable output voltage variation $\Delta V_O = \Delta V_{O,min}$ can be calculated as:

$$\Delta c_{\mathrm{min}} = \left(10^{\frac{\Delta V_{\mathrm{O,min}}}{S_{\mathrm{A}}}} - 1\right) c_{\mathrm{max}} \tag{14}$$

Supplementary References

[1] Bernards, D. A. & Malliaras, G. G. Steady-state and transient behavior of organic electrochemical transistors. *Adv. Funct. Mater.* **17**, 3538–3544 (2007).

[2] Romele, P., Ghittorelli, M., Kovács-Vajna, Z. M. & Torricelli, F. Ion buffering and interface charge enable high performance electronics with organic electrochemical transistors. *Nat. Commun.* **10**, 3044 (2019).

[3] Torricelli, F. *et al*. Transport Physics and Device Modeling of Zinc Oxide Thin-Film Transistors - Part II: Contact Resistance in Short Channel Devices. *IEEE Trans. Electron. Dev*. **58**, 3025-3033 (2011).
